# MiNEN, amphicrine carcinomas, and conventional carcinomas with neuroendocrine differentiation: diagnostic criteria, open questions, and future perspectives

**DOI:** 10.1007/s00428-025-04241-z

**Published:** 2025-09-11

**Authors:** Moritz Jesinghaus, Maxime Philipp Schmitt, Sebastian Foersch, Björn Konukiewitz

**Affiliations:** 1 Institute of Pathology, Philipps-Universität Marburg, Marburg, Germany; 2 Institute of Pathology, University Medical Center Mainz, Mainz, Germany; 3 Institute of Pathology, University Hospital Schleswig-Holstein, Kiel, Germany

**Keywords:** MiNEN, Neuroendocrine carcinoma, Neuroendocrine neoplasm, Neuroendocrine tumor, MANEC, Amphicrine carcinoma

## Abstract

Mixed neuroendocrine and non-neuroendocrine neoplasms (MiNEN) represent a heterogeneous group of bidirectionally differentiated epithelial malignancies that are, in most cases, highly aggressive. They are defined by the presence of morphologically distinct, yet clonally related, neuroendocrine and non-neuroendocrine components, each comprising at least 30% of the tumor mass according to current guidelines. Tumors that fall within the differential diagnostic spectrum of MiNEN include amphicrine carcinomas—characterized by the co-expression of neuroendocrine and non-neuroendocrine features within the same tumor cell—as well as conventional carcinomas that lack neuroendocrine morphology but exhibit immunohistochemical expression of neuroendocrine markers. However, these entities do not fulfill the current diagnostic criteria for MiNEN. In this review, we aim to outline the current diagnostic framework for MiNEN and examine the conceptual and classification boundaries of amphicrine carcinomas and conventional carcinomas with aberrant neuroendocrine marker expression in relation to what is presently defined as a MiNEN. In addition, we highlight key unresolved questions that should be addressed in future guidelines to streamline the diagnostic process and improve consistency. Finally, we provide an outlook on emerging technologies and future perspectives that may further refine the classification and clinical management of these complex neoplasms.

## Introduction

Neuroendocrine neoplasms (NEN) represent a morphologically and molecularly heterogeneous group of tumors [38, 39, 41]. A unifying feature of all NENs is their epithelial origin, combined with characteristic neuroendocrine morphology on hematoxylin and eosin (H&E) staining and the immunohistochemical expression of neuroendocrine markers [2]. NEN comprise two fundamentally distinct entities: well-differentiated neuroendocrine neoplasms (neuroendocrine tumors, NET) and poorly differentiated neuroendocrine neoplasms (neuroendocrine carcinomas, NEC), which are most commonly encountered in their pure forms [13, 15, 38, 39].

However, it is well recognized that a considerable subset of NEN is represented by mixed neoplasms in which morphologically clearly recognizable neuroendocrine and non-neuroendocrine components coexist within the same tumor [20, 21, 33]. These lesions encompass a broad spectrum of possible combinations and are therefore descriptively referred to as mixed neuroendocrine–non-neuroendocrine neoplasms (MiNEN) [3, 21, 41]. The term MiNEN has become established primarily in the context of intestinal pathology, although mixed differentiation of this kind can naturally also occur at extraintestinal sites. However, in other organ systems—such as the female genital tract or the head and neck region—these tumors are not yet explicitly referred to as MiNEN in the respective WHO classifications [40]. The most common form of MiNEN is the combination of an adenocarcinoma with a NEC (mixed adenocarcinoma–NEC), which typically display NEC-like biological behavior and are associated with a worse prognosis compared to conventional carcinomas of the same anatomical site.

Despite this terminological definition, the field of MiNEN remains marked by considerable diagnostic uncertainty and poorly defined grey zones, as several important questions remain unresolved [34, 38, 41]. This presents challenges not only for diagnostic pathologists but also for the clinical disciplines involved in patient management. Key issues include: (1) how to classify tumors that exhibit morphologically recognizable mixed differentiation but fall short of the 30% threshold, (2) how to approach amphicrine neoplasms, which display both lineages within the same tumor cell or closely intermingled populations lacking clear zonal separation, and (3) how to interpret conventional carcinomas with unequivocally non-neuroendocrine morphology on H&E that nonetheless diffusely express neuroendocrine markers.

In this review, we aim to provide a comprehensive overview of the most relevant aspects of mixed neoplasms for diagnostic pathologists. We introduce the conceptual framework of MiNEN and outline practical diagnostic criteria for accurate classification. In the subsequent sections, we examine amphicrine carcinomas and conventional carcinomas with neuroendocrine marker expression, in order to evaluate their current position in relation to what is presently defined as true MiNEN. Finally, we discuss unresolved questions and explore future perspectives.

## Definition and underlying concept of MiNEN

MiNEN represent a broader category of mixed epithelial neoplasms composed of both a neuroendocrine and a non-neuroendocrine component. Each component must be morphologically and immunohistochemically identifiable and contribute substantially to the tumor mass, with an arbitrarily defined cutoff of at least 30%. The term was introduced by the WHO in 2017 to replace previously inconsistent terminology and to provide a framework that reflects the broad spectrum of possible combinations between neuroendocrine and epithelial non-neuroendocrine components [38, 41].

The earlier designation “mixed adenoneuroendocrine carcinoma” (MANEC) had been widely used to describe such lesions, typically referring to the most frequent constellation of an adenocarcinoma combined with a NEC [3, 21]. However, this terminology was too restrictive, as it did not encompass the full histological diversity observed in these neoplasms. MiNEN may also involve other non-neuroendocrine components, such as squamous cell carcinoma or acinar cell carcinoma, and in some cases, the neuroendocrine component may be a NET rather than a NEC. The introduction of the MiNEN concept thus acknowledges this morphological and biological heterogeneity and offers a more inclusive and flexible classification framework.

Accordingly, MiNEN should be regarded as a descriptive term that denotes dual epithelial differentiation and requires further specification by explicitly naming the histological types of both the neuroendocrine and non-neuroendocrine components. The exact composition may vary considerably depending on the anatomical site and biological context of the tumor.

### How to diagnose MiNEN

**Fig. 1:**
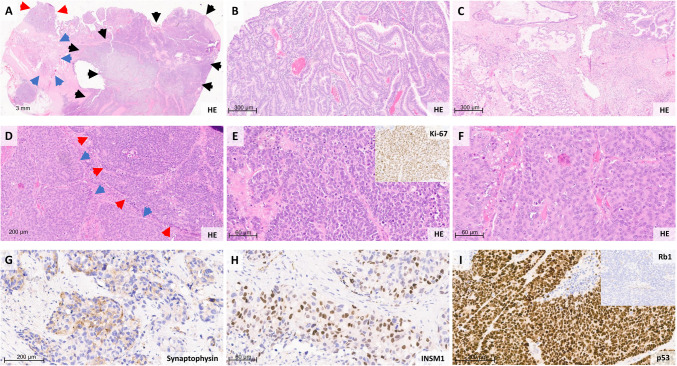
Histological example of a high-grade MiNEN. **A** Scanning magnification of a high-grade MiNEN, specifically a mixed adenocarcinoma–NEC located at the ileocecal valve. The red arrow indicates a tubulovillous adenoma, serving as a shared precursor lesion; the blue arrow marks an area of invasive adenocarcinoma, while the black arrow highlights the NEC component, which in this case exhibited both small-cell and large-cell morphology. **B** Higher magnification of the precursor lesion showing a tubulovillous adenoma (here with low-grade intraepithelial neoplasia). **C** Invasive adenocarcinoma component with focal mucinous differentiation.** D** Transition zone between the small-cell NEC (red arrow) and large-cell NEC (blue arrow), further illustrated in (E) (small-cell NEC, inset: Ki-67) and (F) (large-cell NEC). **G** The NEC component shows heterogeneous expression of synaptophysin and INSM1 (H). **I** Nuclear p53 overexpression of the NEC component (inset: loss of Rb1 expression).* Modified from *[36]

MiNEN are most commonly composed of an invasive non-neuroendocrine carcinoma in combination with a NEC, whereas combinations involving a NET are exceedingly rare [8, 22, 34, 38, 41]. With regard to spatial organization, some MiNEN display a composite or mosaic pattern [23, 43], in which the two components appear mostly separated and occupy distinct but adjacent areas. In contrast, others exhibit an intermingled pattern, characterized by a diffuse and intimate admixture of both components, which nevertheless remain morphologically and immunophenotypically distinguishable. A representative example of a high-grade MiNEN, specifically a mixed adenocarcinoma–NEC is given in Fig. 1.

The distinct identification of both components on H&E staining is the essential first step toward establishing the diagnosis. Once this morphological distinction is made, the next critical step is to determine the degree of differentiation of the neuroendocrine component, as this has major implications for biological behavior and clinical outcome. Poorly differentiated neuroendocrine components are classified as either large cell or small cell NEC, although mixed forms may occasionally occur.

Large cell NECs (LC NEC) are high-grade neoplasms characterized by solid growth, large nuclei with a clearly recognizable nucleolus of variable size, a high mitotic rate (mostly > 20 per 2 mm^2^, though cutoffs may vary depending on the anatomical site) an elevated Ki-67 index above 20%, and/or necrosis. Small cell NECs (SC NEC) consist of small cells with scant cytoplasm, hyperchromatic nuclei, and inconspicuous nucleoli, and likewise exhibit high proliferative activity. In contrast, morphological features suggestive of a well-differentiated neuroendocrine component include monomorphic nuclei with salt-and-pepper chromatin, a considerably lower mitotic activity, and an organoid architectural growth pattern.

To further support the morphological impression of a neuroendocrine component, confirmation by immunohistochemistry is mandatory. NECs should consistently express at least one neuroendocrine marker—such as synaptophysin, INSM1, or chromogranin A—along with a Ki-67 index exceeding 20%, which in most cases is substantially higher and usually easily exceeds 55% [37]. It should be noted that in NECs, particularly those with small cell morphology, expression of neuroendocrine markers may be variable or focal [1, 2, 14, 27, 29, 35]. In NETs, by contrast, strong and diffuse expression of neuroendocrine markers is expected, although chromogranin A may be weak or absent in certain sites, such as the rectum. The Ki-67 index in NETs is typically low and should be used to assign the tumor grade (G1–G3) according to WHO criteria [38, 39, 41].

In cases where the neuroendocrine (NEN) component displays ambiguous morphology and both a NET G3 and a poorly differentiated NEC—typically of the large cell type—may be considered based on conventional histology, selected immunohistochemical stains can aid in the differential diagnosis, provided they are interpreted in conjunction with the tumor’s morphological features and anatomical localization. This panel may include classical surrogate markers for underlying molecular alterations, such as p53 and Rb1, which often display aberrant expression patterns in NEC; Ki-67 as a marker of proliferative activity, with high indices supporting a diagnosis of NEC; differentiation-associated markers such as somatostatin receptors—particularly SSTR2A—which are rarely strongly expressed in NEC; and the expression profiles of broad-spectrum cytokeratins or general neuroendocrine markers, which can be reduced in NEC compared to NET [1, 14–16, 18, 29, 35].

The non-neuroendocrine component of MiNEN is generally represented by conventional carcinomas, most commonly adenocarcinomas, and less frequently squamous cell carcinomas. These should be graded and subclassified according to the histological criteria applicable to the respective primary site. In the pancreas, acinar cell carcinomas represent an important differential diagnosis for the non-neuroendocrine component and must be interpreted with caution. As discussed in more detail below, tumors of this group should only be classified as MiNEN if the H&E morphology clearly shows a biphasic pattern with both acinar differentiation and a distinct neuroendocrine component—resembling either a NET or a NEC—and if the respective components are confirmed by appropriate immunohistochemical markers [15]. Importantly, only mixed neoplasms in which the non-neuroendocrine component is invasive fulfill the diagnostic criteria for MiNEN. The presence of non-invasive precursor lesions—such as adenomas—adjacent to an otherwise invasive NEC does not meet these requirements, and the invasive neoplasm should instead be diagnosed as NEC, while the presence of the precancerous lesion should be mentioned in the pathology report.

Molecular analyses have provided compelling evidence that the two components of MiNEN are clonally related, a finding that aligns with the frequent observation of associated precancerous lesions in these tumors. Moreover, genetic profiling across various sites of the digestive tract has demonstrated that the mutational landscape of high-grade MiNEN (e.g. mixed adenocarcinoma-NEC) shares major molecular alterations with conventional carcinomas of the same anatomical location [7, 10, 11, 16, 42]. However, extended molecular profiling is not required for the diagnosis of MiNEN. Rather, its emerging role lies in identifying potential therapeutic targets that may support individualized treatment strategies for these often clinically challenging neoplasms.

**Fig. 2:**
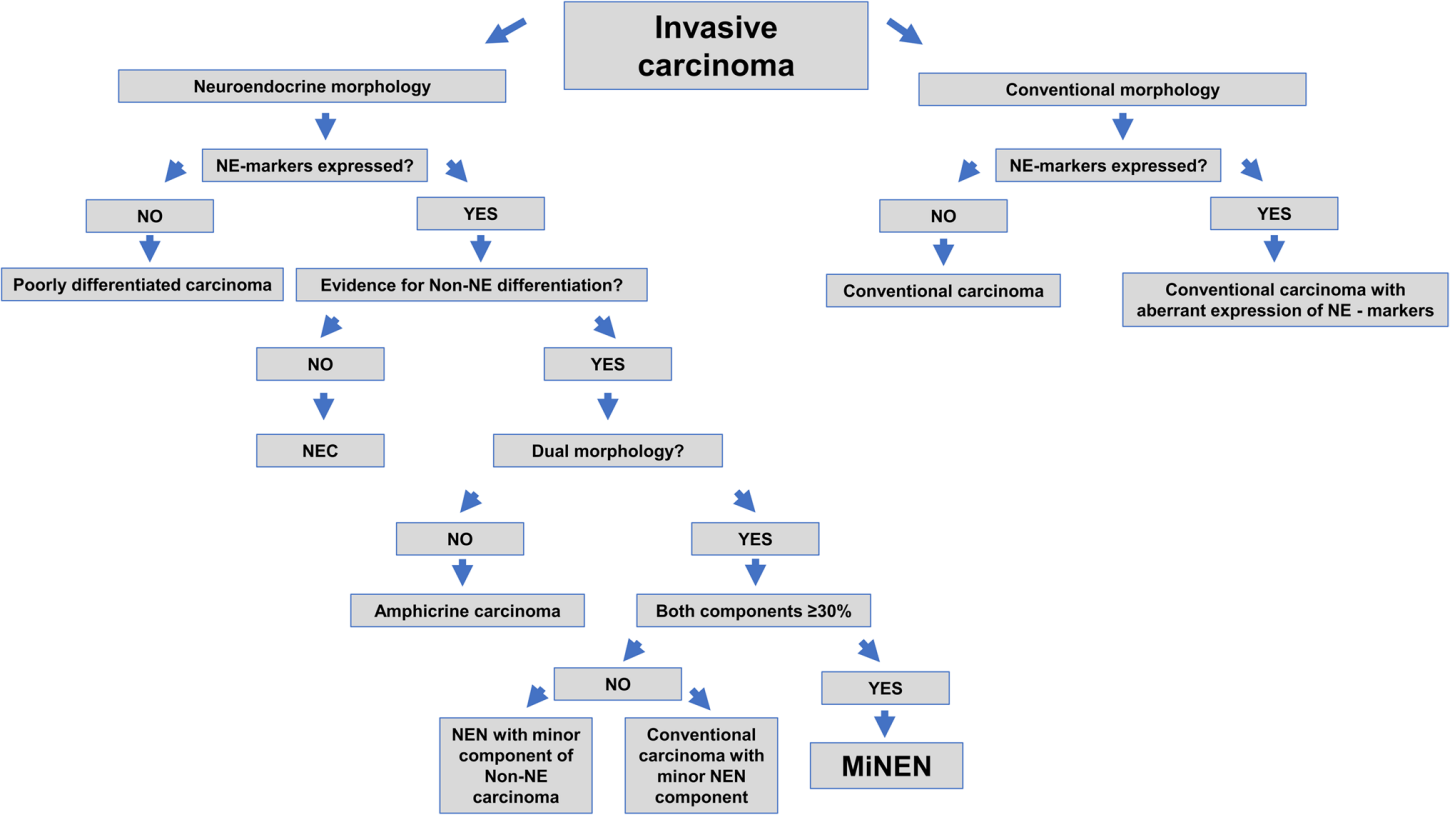
Diagnostic algorithm for the diagnosis of MiNEN and key differential diagnostic considerations. NE markers: Synaptophysin, Chromogranin A, INSM1. Abbreviations: NE, Neuroendocrine; NEN, neuroendocrine neoplasm; MiNEN, mixed neuroendocrine–non-neuroendocrine neoplasm

A comprehensive algorithm for the diagnosis of MiNEN including the most important differential diagnoses is given in Fig. 2.

## What are amphicrine carcinomas?

Amphicrine (or amphicrine-like) carcinomas are rare epithelial neoplasms that display both neuroendocrine and non-neuroendocrine differentiation within individual tumor cells or in a closely admixed cellular population [23, 30, 32, 43]. Their status within the evolving taxonomy of neuroendocrine neoplasms—particularly in relation to MiNEN—remains unresolved, and robust diagnostic criteria have yet to be established.

**Fig. 3:**
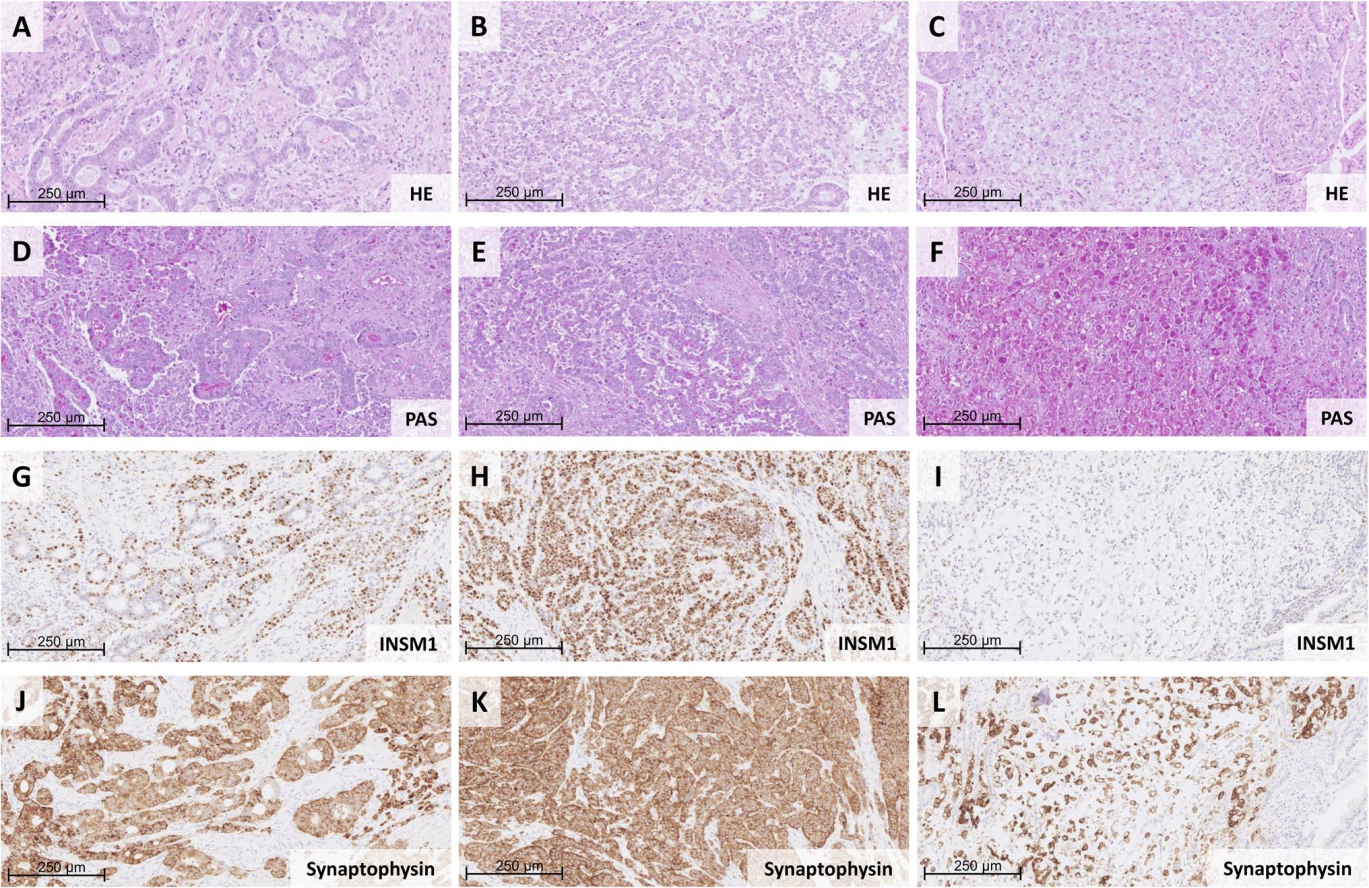
Amphicrine carcinoma showing hybrid morphology**.** The tumor exhibits areas of glandular (**A**), trabecular (**B**), and signet ring cell differentiation (**C**). All components demonstrate amphicrine features, evidenced by strong PAS positivity (**D, E, F**) and co-expression of neuroendocrine markers (**G, H, J, K, L**). The signet ring cell component is strongly positive for synaptophysin (L) but negative for INSM1 (**I**)

Morphologically, amphicrine carcinomas are defined by tumor cells that concurrently exhibit features of both exocrine and neuroendocrine differentiation. This includes, on the one hand, evidence of mucin production or glandular architecture, and on the other hand, features suggestive of neuroendocrine differentiation, such as large vesicular nuclei consistent with large-cell NEC morphology, salt-and-pepper chromatin typical of well-differentiated NETs and/or the presence of neuroendocrine granula. To support the diagnosis, these morphological characteristics have to be accompanied by the expression of neuroendocrine markers such as synaptophysin, chromogranin A, or INSM1. In contrast to MiNEN, amphicrine carcinomas lack a zonal or biphasic architecture. The dual phenotype may be observed within individual tumor cells or as a finely intermingled population of cells with distinct immunophenotypic profiles, but without clear morphological separation. An example of a neoplasm that we would classify as an amphicrine carcinoma is given in Fig. 3. One group that may serve as a prime example of the diagnostic challenges within this category is located in the pancreas—namely, acinar cell carcinomas exhibiting expression of neuroendocrine markers. In contrast to MiNENs, these tumors lack a biphasic architecture by definition but frequently display cytological reminiscent of large cell neuroendocrine carcinoma (LC NEC), particularly vesicular nuclei with prominent nucleoli in cells that otherwise show unequivocal acinar morphology [15, 38]. In our view, the combination of these cytological features with neuroendocrine marker expression may warrant consideration of criteria consistent with true amphicrine differentiation.

Data on the molecular background and the clinical behavior of amphicrine carcinomas remain scarce. The most informative studies to date have focused on gastric tumors and report a marked predominance of male patients. Whole-exome sequencing has revealed frequent somatic mutations in common carcinoma-associated genes such as *TP53* and *APC*, but no mutation pattern that reliably distinguishes amphicrine carcinomas from true MiNEN. Transcriptomic profiling suggested a molecular proximity to adenocarcinomas [32].

Future studies based on well-characterized morphological cohorts of amphicrine carcinomas are needed to clarify their position in relation to non-neuroendocrine carcinomas, NETs, MiNEN, and pure NECs. This includes a more precise delineation of robust histological criteria, a better understanding of their molecular pathology, and—most importantly—an improved assessment of their prognostic relevance and response to treatment regimens.

In the absence of more comprehensive data from systematically characterized cohorts, we support the notion that amphicrine carcinomas should—at least for now—not be subsumed under MiNEN. In routine diagnostic practice, we advocate that amphicrine carcinomas be diagnosed as such, accompanied by a comment stating that they appear to represent a distinct entity within the neuroendocrine neoplasm spectrum, although robust data regarding their biological behavior and molecular characteristics remain limited.

## Conventional carcinomas with expression of neuroendocrine markers: clinically significant?

**Fig. 4:**
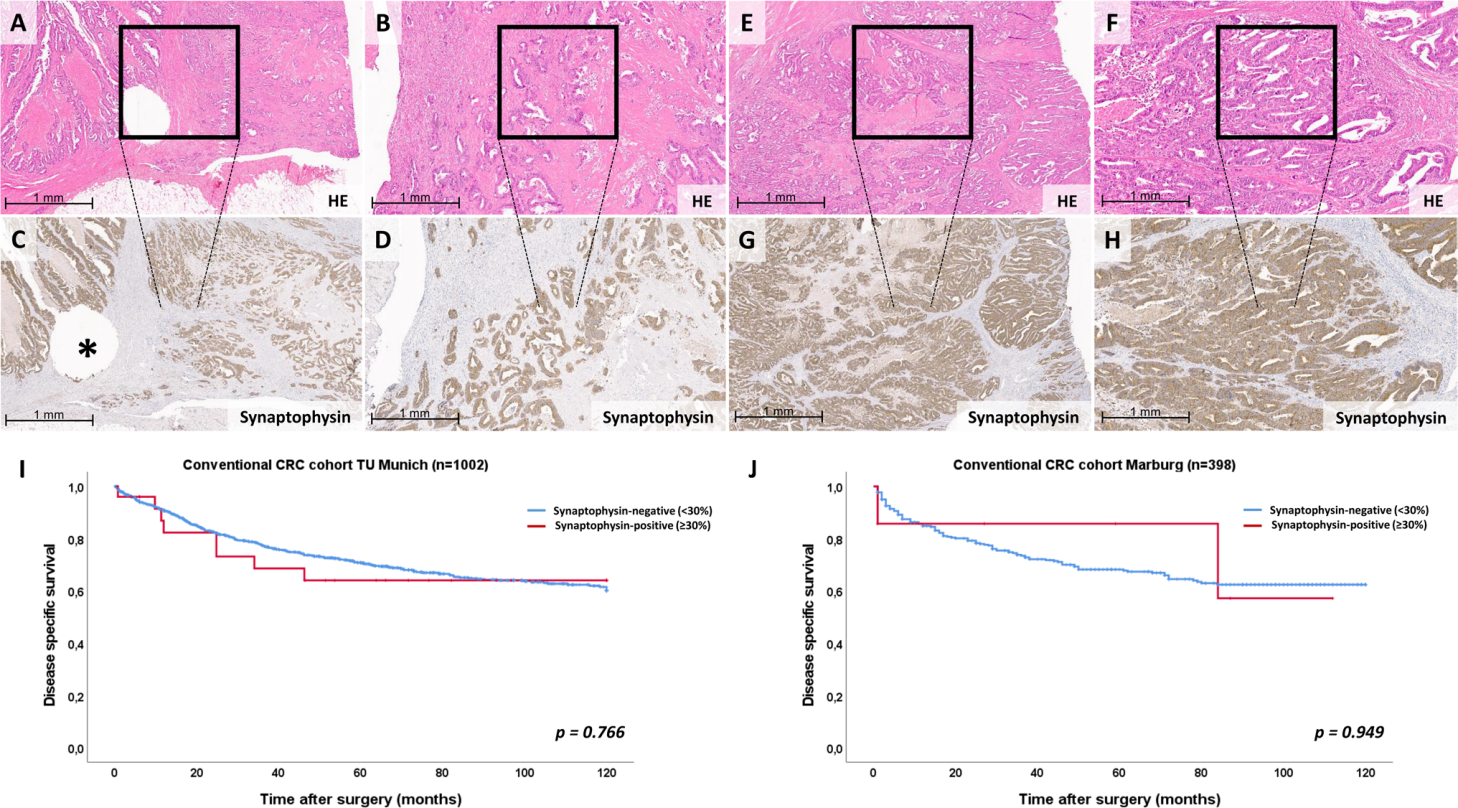
Expression of neuroendocrine markers in conventional carcinomas.** A, B, E, F** H&E staining of two conventional colorectal adenocarcinomas NOS, each displaying purely glandular architecture without any morphological features consistent with a small-cell or large-cell neuroendocrine carcinoma component. **C, D, G, H** Synaptophysin staining of the corresponding regions from these same tumors reveals diffuse synaptophysin expression despite the absence of neuroendocrine morphology. **I** No prognostic impact is observed for diffuse synaptophysin expression (defined here as ≥30%) in morphologically non-neuroendocrine colorectal carcinomas, based on a cohort of 1002 cases. **J** Similar results were obtained in an independent validation cohort of 398 colorectal carcinomas reviewed specifically for the purpose of this study.* Modified from Konukiewitz *et al*. Cancers, 2021*

Another group of neoplasms whose position within the spectrum of neuroendocrine neoplasms—and particularly in relation to MiNEN—remains a subject of ongoing debate are conventional carcinomas with diffuse expression of neuroendocrine markers, most commonly represented by adenocarcinomas, and less frequently by squamous cell carcinomas. We would define these as carcinomas that exhibit the typical morphology of a conventional carcinoma without characteristic neuroendocrine features on H&E staining, yet show diffuse (typically isolated) immunohistochemical expression of one neuroendocrine marker, especially synaptophysin, exceeding the commonly applied 30% threshold. In two colorectal cancer cohorts studied by our group, the frequency of such tumors was approximately 1%, although a recent study reported a frequency of 4% [17, 24]. Although the WHO classification emphasizes the requirement for distinct neuroendocrine morphology in the diagnosis of MiNEN, testing for neuroendocrine markers in an unequivocally non-neuroendocrine neoplasm can place the diagnostic pathologist in a diagnostic dilemma. This approach is explicitly discouraged by the WHO for certain organ sites such as the lung or the breast [38, 39, 41]. In previous studies conducted by our group on more than one thousand conventional colorectal carcinomas (adenocarcinoma NOS and variants) from the University Hospital of the Technical University of Munich, we found no prognostic relevance associated with the expression of either synaptophysin or INSM1. In contrast, true MiNEN (mixed-adenocarcinoma-NEC) and NEC were associated with markedly inferior survival [17, 24]. As part of this review, we re-evaluated this finding using the same methodology as described previously [17] in an independent validation cohort of 398 conventional colorectal carcinomas from the University Hospital Marburg with respect to synaptophysin expression. Likewise, we observed no association with patient outcome. Similar findings have been reported for pulmonary carcinomas and are also consistent with the narrative of previous reviews on MiNEN [9, 12, 19, 31, 34]. Figure 4 illustrates representative examples of what we define as conventional adenocarcinomas exhibiting aberrant expression of neuroendocrine markers (Synaptophysin), and demonstrates the lack of prognostic impact of this rare phenomenon. This conclusion is supported by data from our previously published studies [17, 24] as well as by an independent validation performed for this review in a separate cohort.

However, it must be noted that findings in the literature are not uniform, as a recent study has suggested a potentially significant prognostic impact of this aberrant neuroendocrine differentiation in colorectal cancer [28]. Therefore, future studies are warranted not only to clarify the situation in colorectal cancer, but also to systematically address the prognostic relevance of neuroendocrine marker expression in morphologically non-neuroendocrine carcinomas arising from other intestinal and extraintestinal sites.

## Unsolved issues in MiNEN

One of the most critical issues in the diagnosis of MiNEN is the arbitrarily defined 30% cutoff, which is currently required for both components. We view this threshold with caution for several reasons. To our knowledge, it has not yet been validated in studies as the most prognostically meaningful threshold in resection specimens. Furthermore, it raises the practical question of how to formulate a diagnosis on biopsy specimens of tumors with dual morphology, as these typically sample only a limited portion of the lesion. In such cases, the diagnosis may shift toward a conventional carcinoma with a focal neuroendocrine component or, conversely, a neuroendocrine neoplasm with a minor non-neuroendocrine fraction once the resection specimen is available. This concern, of course, does not apply to cases where dual morphology is not captured on the initial biopsy and where a diagnosis of MiNEN can only be made upon examination of the resection specimen.

Given that most MiNEN contain NEC components, which are typically the main drivers of biological aggressiveness, we would advocate that any tumor showing clear dual morphology of the invasive components on H&E staining should be considered eligible for future categorization as MiNEN, even if the respective proportions fall below the 30% threshold. In contrast, we would rather advocate against inclusion of mixed lesions with a precancerous lesion (e.g., an adenoma combined with a NEC or a NET) into the MiNEN category, as we believe the nomenclature of the disease should reflect the invasive components, which are the harmful ones for the patients [8, 22]. In our view, the nomenclature should reflect the invasive components of the neoplasm, as these determine the biological behavior and clinical relevance for the patient.

Another important aspect that we would advocate for in future classifications is the highest possible degree of harmonization in terminology and diagnostic cutoffs across different organ systems—particularly between the gastrointestinal tract and thoracic organs, where the vast majority of NENs and mixed tumors with neuroendocrine components occur. In the lung, the term *combined carcinoma* refers to mixed neoplasms and is not restricted to those with both neuroendocrine and non-neuroendocrine components [39]. For example, it also includes tumors with dual neuroendocrine morphologies, such as mixtures of small cell and large cell neuroendocrine carcinoma. Combined small cell lung carcinomas (SCLC) are defined as tumors composed of an SCNEC component in combination with one or more non-small cell components. These additional elements may include LC NEC, large cell carcinoma (LCC), adenocarcinoma, squamous cell carcinoma (SCC), or spindle/giant cell carcinoma. For combined SCLC with LCNEC or LCC, the non-small cell component must comprise at least 10% of the tumor; this quantitative criterion does not apply to adenocarcinoma or SCC components. Combined LCNECs most frequently occur in combination with adenocarcinoma, although other non-neuroendocrine variants may also be present. No clearly defined cutoff is defined for these combinations. As emphasized above, we strongly advocate for the implementation of a unified and consistent terminology for mixed neoplasms and NENs across all organ systems, ideally supported by standardized quantitative thresholds. Where differences between organ systems are retained, they should be explicitly justified by robust evidence demonstrating a clear prognostic relevance or diagnostic benefit.

Furthermore, as already outlined in the respective sections above, the prognostic relevance and biological standing of amphicrine carcinomas and morphologically non-neuroendocrine carcinomas with aberrant expression of neuroendocrine markers should be clearly defined in relation to true MiNEN. While further studies are needed to elucidate the molecular underpinnings and clinical behavior of amphicrine carcinomas—and to assess whether their inclusion in the MiNEN category may eventually be justified—we currently argue against an inclusion at this timepoint. Nonetheless, we acknowledge that amphicrine carcinomas constitute a morphologically and biologically distinct entity, and we believe that this concept should be further refined and formally established as more data become available.

In contrast, we oppose classifying morphologically non-neuroendocrine carcinomas that merely express neuroendocrine markers as MiNEN, and we would also caution against the use of neuroendocrine immunohistochemical stains in the absence of corresponding morphological features. The expression of neuroendocrine markers alone does not reliably indicate functional neuroendocrine differentiation, nor does it consistently correlate with clinical behavior. Moreover, data on distinct molecular profiles or treatment responses in such cases remain lacking. Aberrant marker expression may instead reflect epigenetic dysregulation, clonal evolution, or nonspecific antigenicity—rather than a true dual-lineage neoplasm, which, in our view, requires a distinct and reproducible neuroendocrine morphology. Including such cases under the MiNEN umbrella would risk diluting the definition, introducing diagnostic ambiguity, and ultimately compromising prognostic assessment and therapeutic decision-making. Nevertheless, further studies on this topic are clearly warranted.

## Future perspectives

The rapid pace of technological development offers tremendous opportunities to deepen our knowledge and understanding of MiNEN and to fundamentally change the way we view these complex neoplasms. One striking example from the past decade is the advancement of sequencing technologies, which has significantly improved our insight into MiNEN biology. These studies have not only confirmed that the dual components of MiNEN are clonally related and arise from a common progenitor cell, but have also clearly demonstrated that the genetic profile of high-grade MiNEN and NECs closely resembles that of conventional carcinomas at the respective anatomical site and is distinct from that of NETs [7, 10, 11, 16, 42].

MiNEN are a prime example of histological heterogeneity, as this feature is inherent to their very definition. Some important questions that remain unanswered are whether the morphological heterogeneity observed in MiNEN is (1) associated with distinct gene expression profiles, and (2) restricted to morphologically distinguishable tumor areas—or whether transcriptional heterogeneity also exists within morphologically uniform components. Addressing these questions will be key to understanding the biological basis of phenotypic divergence in MiNEN and may help refine diagnostic criteria and therapeutic strategies. One technology that may be particularly valuable in this context is spatial transcriptomics (ST)—a comparatively novel method that enables the visualization and quantification of gene expression within intact tissue sections while preserving spatial context [4, 26]. By integrating histological architecture with transcriptomic data, ST allows researchers to spatially map gene expression patterns and identify transcriptionally distinct subregions within tumors. Exploratory data from our group on high-grade MiNEN, specifically mixed adenocarcinoma-NEC [36], suggest an even greater degree of transcriptional heterogeneity than previously anticipated, including within morphologically uniform tumor regions, which also showed differences in their predicted chemotherapy response.

We believe that novel technologies such as spatial transcriptomics (ST), along with others like proteomics—which likewise holds great potential—will be essential for linking morphological phenotypes to underlying biology and enabling spatially informed molecular profiling in complex neoplasms such as MiNEN. This is particularly relevant as these technologies are expected to become increasingly precise, scalable, and clinically applicable in the near future, thereby enhancing their overall utility in both diagnostic practice and research.

As in many areas of medicine—and arguably across all fields of science—great promise lies in the application of artificial intelligence (AI) and computational pathology [6], which may also prove highly beneficial for diagnostic pathologists working in the field of MiNEN. Currently, pathology foundation models are trained on various diagnostic, prognostic, and predictive tasks with highly encouraging results [5, 25]. However, no such tools are available for NEN to date. AI-powered tools may have the potential to support not only the reliable detection and classification of MiNEN and NENs in general, but also the differentiation from the previously discussed key diagnostic mimics, such as distinguishing NET from NEC, or MiNEN from amphicrine carcinoma and conventional carcinomas with aberrant neuroendocrine marker expression. Furthermore, AI-based image analysis may assist in accurately quantifying the relative proportion of each tumor component within a mixed neoplasm—an essential criterion for establishing the diagnosis of MiNEN.

Beyond these diagnostic applications, future AI models may also aid in the prediction of therapeutically relevant molecular alterations, particularly in the context of histological tumor heterogeneity. Moreover, such tools may contribute to more refined prognostic assessment and enable personalized predictions of treatment response, both to conventional and targeted therapies [6].

## Conclusions

As our understanding of MiNEN continues to evolve, it becomes increasingly clear that current diagnostic frameworks—while improved compared to earlier versions—still require nuanced conceptual refinement and do not yet fully capture the biological complexity and clinical diversity of these tumors. Evidence-based harmonization of diagnostic cutoffs and terminology across organ systems may represent an important step forward. Furthermore, the integration of rapidly emerging technologies such as spatial transcriptomics or AI-driven computational pathology holds great promise for the future.
